# Metabolite Profiling for Typization of “Rucola della Piana del Sele” (PGI), *Eruca sativa*, through UHPLC-Q-Exactive-Orbitrap-MS/MS Analysis

**DOI:** 10.3390/foods12183384

**Published:** 2023-09-09

**Authors:** Maria Assunta Crescenzi, Antonietta Cerulli, Paola Montoro, Sonia Piacente

**Affiliations:** 1 Department of Pharmacy, University of the Study of Salerno, Via Giovanni Paolo II, 132, I-84084 Fisciano, SA, Italy; mcrescenzi@unisa.it (M.A.C.); acerulli@unisa.it (A.C.); piacente@unisa.it (S.P.); 2 PhD Program in Drug Discovery & Development, Department of Pharmacy, University of the Study of Salerno, Via Giovanni Paolo II, 132, I-84084 Fisciano, SA, Italy

**Keywords:** typization, metabolite profiling, protected geographical indication

## Abstract

In August 2020, the *Eruca sativa* cultivar “Rucola della Piana del Sele” obtained from the European Union the prestigious PGI (protected geographical indication) label, which certifies the uniqueness of its characteristics and increases its prestige both nationally and, above all, internationally. This plant is recognized as a product of excellence, with a unique flavor and unmistakable aroma. Therefore, since there are no methods to characterize the PGI product, a metabolomic approach was applied to characterize *E. sativa* grown in the Piana del Sele and different geographical areas. As *E. sativa* has very wide cultivation, this study sought to compare the metabolite profiles of rocket grown in Piana del Sele, Bergamo, and Brescia, as well as in Switzerland, making a comparison also with the metabolite profile of *E. sativa* grown spontaneously. To determine the best procedure to distinguish “Rucola della Piana del Sele” from the others, different extraction procedures were carried out using different solvents and fresh or freeze-dried plant matrices. The different extracts were analyzed by liquid chromatography coupled with high-resolution mass spectrometry experiments, using chemometric analyses to identify biomarker metabolites that characterize the PGI product. The LC-ESI-Q-Exactive-MS/MS profiles of methanol and hydroalcoholic extracts of different cultivars of *E. sativa* were found to be rich in bioactive compounds such as glucosinolates, glycosylated flavonoids, fatty acids, and lipids. The LCMS data were analyzed by principal component analysis (PCA); the score scatter plot shows significant separation among *Eruca* samples grown in different geographical areas. In detail, loading the scatter plot revealed *Eruca* grown in Piana del Sele to be richer than other cultivars in glycosylated quercetin 3,3′,4′-*O*-triglucoside (**7**), quercetin-3,4′-*O*-diglucoside-3′-*O*-(6-sinapoyl-glucoside) (**10**), and quercetin diglucoside (**30**). Finally, considering the biological interest in erucin, the myrosinase product of glucoerucin, the latter was quantified in the extracts by LC-ESI/QTrap/MS/MS using the multiple reaction monitoring (MRM) method; *E. sativa* from Piana del Sele showed the highest content of glucoerucin.

## 1. Introduction

*Eruca sativa*, also known as rocket salad, is an annual herbaceous plant belonging to the Brassicaceae family. It is distributed worldwide [1] and is very popular worldwide for its edible green leaves, commonly used in salad and food dish recipes. Asia and the Mediterranean region are the places of maximum bioavailability. What mainly distinguishes this plant are its leaves, with their typical characteristic smell and pungent taste [2].

The leaves of *E. sativa* are largely used in the food sector. They are eaten raw in salads or even cooked in different ways, e.g., to prepare pestos.

*E. sativa* has beneficial properties in the prevention and treatment of cancer, together with cardiovascular diseases, including atherosclerosis and thrombosis. It is reported to also possess antibacterial, antiviral, antioxidant, antidiabetic, anti-inflammatory, and immunomodulatory activities [3,4,5,6].

*Eruca* is rich in mineral salts and vitamins such as vitamins A, K, E, B_5_, and C. Due to its richness in vitamin C, it can stimulate the immune system [7].

Its main class of secondary metabolites, mainly contained in the leaves and seeds, is represented by glucosinolates [8]. These metabolites are hydrolyzed by the enzyme myrosinase, producing bioactive products such as nitriles, thiocyanates, and isothiocyanates [9,10].

Isothiocyanates have shown antiproliferative activity in human lung cancer cells, hepatoma cells, colon cancer cells, prostate cancer cell lines, and leukemia cells [11]. A paper published recently showed the activity of glucosinolates isolated from *E. sativa* and their hydrolysis products in the treatment of breast cancer, the most frequent form of cancer occurring in women of any age, and particularly against triple-negative breast cancer (TNBC) with a mechanism involving intracellular ROS (reactive oxygen species) [12]. General interest in these plants was enhanced in recent years by a direct interest in erucin, 4-(methylthio)butyl isothiocyanate (ER), which is a new potential chemopreventive agent present in rocket species, whose pungent flavor is partially associated with this volatile isothiocyanate [13].

In addition, among the various isothiocyanates, erucin showed slow and long-lasting release of H_2_S. In recent studies, erucin demonstrated vasorelaxing and anti-hypertensive properties in in vitro and in vivo experimental models. Moreover, it also exhibited the ability to protect human endothelial and aortic smooth muscle cells against the damage induced by high glucose levels [14,15].

Erucin is not directly present in *E. sativa*, but it is a product of myrosinase on glucoerucin, a glucosinolate present at high levels in the species. 

The *Eruca* grown in the Piana del Sele (Salerno, Italy) received the prestigious PGI label in August 2020 and has become one of the flagship products of the Piana del Sele, so much so that it can be defined as the “green gold” of the area.

The typical characteristics of this product, with its intense green color and unmistakable shape, have given it an inestimable and inimitable value. In particular, the nutritional characteristics of the PGI rocket from Piana del Sele are the result of cutting-edge cultivation methods that respect tradition.

These techniques, in fact, allow the “Rucola della Piana del Sele” to grow luxuriantly and to acquire, one by one, all the typical organoleptic and nutritional characteristics of this unique product.

The influence of culture systems and harvest times on the specialized metabolite-based chemical composition of *E. sativa* leaves is not entirely understood. A paper published recently by Buitrago-Villanueva et al. evaluated the influence of the culture system and harvest time on the specialized metabolite composition, reporting that some compounds were relevantly accumulated by the effect of soilless cultures. An LC-MS analysis combined with a multivariate data analysis was selected as the election platform for these specific analyses [16].

The same effect on metabolism is to be expected based on the influence of geo-climatic aspects. Until today, no methods have been reported to type and characterize the “Rucola della Piana del Sele”. For this reason, the objective of this research was to determine the best extraction and characterization technique to distinguish the PGI product from *Eruca* plants coming from other geographical areas, based on specialized metabolite composition.

The analytical approach to the phytochemical composition of *E. sativa* over the last ten years has been mainly explored by LC-MS [17,18], sometimes followed by a chemometric approach [16].

In the present work, different extracts obtained from different cultivars of *E. sativa* and under different extraction methods were examined through a metabolite profiling approach by using liquid chromatography coupled with high-resolution mass spectrometry equipped with an electrospray source (UHPLC-Q-Exactive-Orbitrap-MS/MS) in negative ion mode.

The multivariate data analysis, released by principal component analysis (PCA), allowed us to process the obtained results and to identify the biomarker metabolites of PGI *E. sativa.*

Extracts of *E. sativa* were found to be rich in polyphenolic compounds, glucosinolates, oxylipins, and lipids. Metabolite profiling of hydroalcoholic extract of freeze-dried *E. sativa* was useful for the typization of “Rucola della Piana del Sele”.

Following a comprehensive metabolomics approach, a targeted approach was then applied to confirm the presence of glucoerucin (the GLS precursor of erucin) and to evaluate the quantitative content of this metabolite in “Rucola della Piana del Sele” in comparison with *E. sativa* from different geographical origins. 

Quantitative analysis of glucosinolates from *E. sativa*, as well as from other plants pertaining to the Brassicaceae family, was intensively reported in literature in recent years, mainly by using HPLC-MS targeted approaches [19,20,21].

In order to quantify glucoerucin in samples under investigation in the present paper, a new quantitative method was developed by UHPLC-Q-Trap-MS/MS in multiple reaction monitoring with enhanced selectivity and sensitivity. The method was then validated according to the European Medicines Agency’s guidelines (EMA quality guidelines ICH Q2).

## 2. Materials and Methods

### 2.1. Raw Materials

The PGI “Rucola della Piana del Sele” was supplied by Analisis S.r.l. (Angri, Salerno, Italy). The *E. sativa* samples grown in Bergamo, Brescia, and Switzerland were purchased at sale points where the geographical origin of the vegetable matrices is guaranteed. The *E. sativa* sample growing spontaneously was harvested in Agro Nocerino Sarnese. Based on the different geographical areas where the samples were collected, they were classified as follows: PS—*E. sativa* from Piana del Sele, BE—*E. sativa* from Bergamo, BR—*E. sativa* from Brescia, SW—*E. sativa* from Switzerland, SP—spontaneous *E. sativa*.

### 2.2. Reagents and Solvents

Ethanol, methanol, and water used for the extractions were purchased from VWR (Milan, Italy). Acetonitrile (ACN), formic acid, water, and methanol of LC-MS grade were purchased from Merck (Merck KGaA, Darmstadt, Germany).

### 2.3. Sample Preparation

Different types of extraction were carried out. Firstly, the aerial parts of the plant were extracted both fresh and fresh-dried, having been previously frozen at −80 °C. Two extractions were set up with both types of matrices, one with a methanol solution and the other with an ethanol/water solution (70/30), both assisted by the ultrasonic bath.

Regarding the extraction from the fresh matrix, 2 g of *Eruca sativa* was used and extracted with 20 mL of either methanol or a hydroalcoholic solution (7:3) for 15 min.

Extraction from the freeze-dried matrix was performed by placing 1 g of *Eruca sativa* in 40 mL of methanol or a hydroalcoholic solution (7:3) for 15 min.

The extractions were repeated three times, filtering the extracts with filter paper.

### 2.4. LC-Q-Exactive-Orbitrap/MS and LC-Q-Exactive-Orbitrap/MS/MS Analysis

The extracts thus obtained from different cultivars of *E. sativa* were examined through a metabolite profiling approach by using liquid chromatography coupled with mass spectrometry equipped with an electrospray source (UHPLC-Q-Exactive-Orbitrap-MS/MS). The experiments were executed with a Thermo Scientific liquid chromatography system, equipped with a quaternary Accela 600 pump and an Accela autosampler, combined with a Quadrupole-Orbitrap hybrid mass spectrometer (Q-Exactive Hybrid Quadrupole-Orbitrap, Thermo Fisher Scientific, Bremen, Germany) equipped with an electrospray ionization (ESI) source. A Kinetex EVO C18 5 μm (150 mm × 2.1 mm) column (Phenomenex Aschaffenburg, Germany) was used to perform the separation. The mobile phases employed were water + 0.1% formic acid (A) and acetonitrile + 0.1% formic acid (B). An increasing linear-gradient (*v*/*v*) at a flow rate of 0.200 mL/min of solvent B was used: 0–23 min, from 5 to 40%; 23–45 min, from 40 to 95%; and then back to 5% for 10 min. The mass spectrometer operated in negative ion mode and 10 μL of each sample was used for injection. ESI source parameters were the following: ion source temperature 300 °C; sheath and auxiliary gas flow (N_2_), 50 and 10; sweep gas 0. The full range *m*/*z* adapted to the acquisition of MS spectra was 150–1400. For the fragmentation study, a data-dependent scan was set up through which the precursor ions corresponding to the most intensive peaks were fragmented in the LC–MS analysis with a collision energy of 30%. Xcalibur software version 2.2 was used for instrument control, data acquisition, and data analysis.

### 2.5. Multivariate Data Analysis

To get a better overview of the data, a multivariate statistical analysis was performed to identify the most appropriate extraction system for typing the “Rucola della Piana del Sele”. The LC-Q-Exactive-Orbitrap/MS chromatograms in negative ion mode were analyzed using the free software package MZmine 2.10 (http://mzmine.sourceforge.net/ (accessed on 23 November 2022)), excluding noise from the LC-MS profiles. The data with intensity less than 5.0 × 10^6^ were excluded. Manual peak selection was then performed, yielding 241 peaks for the methanolic extracts of freeze-dried *Eruca*, 350 peaks for the hydroalcoholic extracts of freeze-dried *Eruca*, 379 peaks for the methanolic extracts of fresh *Eruca*, and 143 peaks for the hydroalcoholic extracts of fresh *Eruca*. The software MZmine 2.10 generated a data matrix in table format (.cvs file). A multivariate data analysis was performed using SIMCA P+ software 12.0 (Umetrix AB, Umea, Sweden) for the principal component analysis (PCA). The PCA was performed to define a homogeneous cluster of samples by using the peak area from the LC-MS analysis. Pareto scaling was used to normalize the data before the multivariate data analysis.

### 2.6. LC-ESI/QTrap/MS/MS Analysis: Calibration and Quantification

A quantitative analysis was performed on an LC-ESI/QTrap/MS system, operating in multiple reaction monitoring (MRM) mode [22], on a C18 reversed-phase (RP) column (50 mm, 2.1 mm; Luna Omega C18, 1.6 µm; Phenomenex, Aschaffenburg, Germany) kept at 30 °C, with a flow rate of 0.30 mL/min. A mobile phase consisting of a combination of A (0.5% HCOOH in water, *v*/*v*) and B (0.5% HCOOH in acetonitrile, *v*/*v*) was used. A linear gradient was performed as follows: from 5 to 24% B in 0.9 min, from 24 to 50% B in 0.8 min, from 50 to 80% B in 1.5 min, from 80 to 100% B in 1.0 min, held at 100% B for 1.0 min, and from 100 to 5% in 1.7 min. The autosampler was set to inject 5 µL of extract (2.0 mg/mL).

Stock solutions (1 mg/mL) of GER as the external standard (ES) were prepared by dissolving GER in a solution of methanol. Each stock solution was diluted with appropriate amounts of methanol to obtain seven solutions of different ES concentrations (0.1, 0.25, 0.5, 1.0, 2.5, 5, and 10 µg/µL). Calibration curves were constructed by injecting 5 µL of each standard solution at each concentration level in triplicate. A linear regression analysis was performed using the Analyst 1.6.2 software provided by the manufacturer (Sciex. Milan, Italy). To validate the LC-ESI/QTrap/MS/MS method, precision (at seven concentrations for each compound), specificity, linear range, limit of detection (LOD), and limit of quantification (LOQ) were evaluated according to the previously described procedure [23]. The limit of quantification (LOQ; equivalent to sensitivity) was evaluated by injecting a series of increasingly diluted standard solutions until the signal-to-noise ratio was reduced to 10. The limit of detection (LOD) was estimated by injecting a series of increasingly diluted standard solutions until the signal-to-noise ratio was reduced to 3 [24]. The LOD was 0.05 ng/mL, and the LOQ was 0.016 ng/mL. Moreover, the following parameters were settled for GER: a declustering potential (DP) of −115 eV, an entrance potential (EP) of −12 eV, a collision energy (CE) of 30%, and a collision cell exit potential (CXP) of −9 eV. In this way, a calibration curve is analyzed by linear regression (y = 50.1 x + 8.71 e^3^, R^2^ = 0.996).

## 3. Results

### 3.1. UHPLC-Q-Exactive-Orbitrap-MS and UHPLC-Q-Exactive-Orbitrap/MS/MS Analysis

Since the extractions with methanol or with a hydroalcoholic solution gave similar results, only the data obtained by hydroalcoholic extraction from fresh and freeze-dried matrices are presented.

The LC-ESI-Q-Exactive-MS/MS profiles of the different Eruca extracts were analyzed by Xcalibur 2.2 software from Thermo Fisher Scientific (, which generated accurate masses to be used for the identification of compounds with suitable confidence (ppm ≤ 5). Fragmentation analyses (MS/MS), followed by research in specific databases of natural product spectral data banks such as MassBank (http://www.massbank.jp/Search) and in the literature, showed no relevant differences between the samples obtained by extraction with MeOH or EtOH/H_2_O solutions.

Overall, the samples were found to be rich in bioactive compounds such as glucosinolates (**1**, **3**, **4**, **13**–**15**, **35**), glycosylated flavonoids (**6**–**9**, **11**, **12**, **15**, **36**), fatty acids (**17**, **21**, **22**, **30**, **31**), and lipids (**27**–**29**, **33**, **34**).

**Figure 1:**
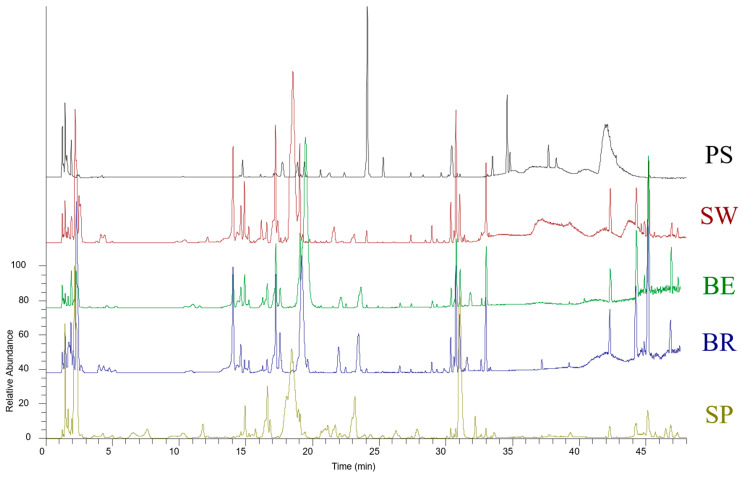
Negative profiles of freeze-dried *E. sativa* ethanolic extracts by UHPLC-Q- Exactive-Orbitrap-MS/MS analysis: PS (Piana del Sele), BE (Bergamo), BR (Brescia), SP (Spontaneous), and SW (Switzerland).

The hydroalcoholic extraction from a freeze-dried matrix was more suitable for typing the Eruca from Piana del Sele, as more characteristic metabolites of the PGI rocket could be identified (Figure 1 and Table 1).

Oxylipins (**17**, **22**, **30**, and **31**) were metabolites identified mainly in the “Rucola della Piana del Sele”.

Compounds **27**–**29**, **30**, and **31** were identified as lipids and were detected in the hydroalcoholic extracts of freeze-dried *E. sativa* samples of rocket grown exclusively in Bergamo, Brescia, and Switzerland. These compounds presented a characteristic fragmentation pattern, where the RCOO fatty acyl ion at *m*/*z* 277 is present as a fragment ion [25] (Figure 1, Table 1 and Table 2).

**Table 1:** Metabolites identified in freeze-dried *E. sativa* ethanolic extracts by UHPLC-Q-Exactive-Orbitrap-MS/MS analysis: PS (Piana del Sele), BE (Bergamo), BR (Brescia), SP (Spontaneous), and SW (Switzerland).

N°	RT	M-H^−^	Molecular Formula	Δppm	MS/MS	Identity	PS	BE	BR	SP	SW	References
**1**	2.23 *	436.04120	C_12_H_22_O_10_NS_3_	2.7	96.9589/178.0173	glucoraphanin		+	+	+	+	[26,27]
**2**	4.21	315.07291	C_13_H_15_O_9_	5.9	152.0106/108.0205	gentisic acid glucoside	+		+			[28]
**3**	7.85	406.03148	C_11_H_20_O_9_NS_3_	5.0	96.9589	glucoiberverin				+		[17]
**4**	12.53	420.04688	C_12_H_22_O_9_NS_3_	4.2	96.9489	glucoerucin				+		[29]
**5**	13.99 *	385.11371	C_17_H_21_O_10_	2.0	205.0499	sinapic acid glucoside		+	+		+	[29]
**6**	14.56 *	433.20758	C_20_H_33_O_10_	1.7	161.04444/101.0230	apigenin glucoside		+	+		+	[29]
**7**	14.76	787.19458	C_33_H_39_O_22_	2.3	301.0349/463.0893	quercetin 3,3′,4′-triglucoside	+	+	+	+	+	[26,27]
**8**	16.16	625.14197	C_27_H_29_O_17_	2.4	301.0349/463.0883	quercetin diglucoside					+	[26,27]
**9**	16.57 *	5631	C_28_H_31_O_17_	1.1	313.0353	isorhamnetin-3,4-diglucoside		+	+	+	+	[26,27]
**10**	17.02	583.05908	C_16_H_27_O_15_N_2_S_3_	1.5	96.9589/178.0173/406.0308	unknown				+		
**11**	17.22	993.25366	C_44_H_49_O_26_	3.0	301.0359/436.0893	quercetin-3,4′-diglucoside 3′-(6-sinapoyl glucoside)	+	+	+		+	[26,27]
**12**	17.53 *	609.14612	C_27_H_29_O_16_	1.8	301.0347	rutin		+	+			[30]
**13**	17.54	477.06488	C_17_H_21_O_10_N_2_S_2_	3.6	96.9589/275.9957	neo			+			[17]
**14**	17.74	405.02328 [2M-H]^−^	C_22_H_39_N_2_O_18_S_6_	3.2	96.9589	DMB-GLS	+	+	+	+	+	[17]
**15**	18.88	1199.31042	C_55_H_59_O_30_	1.5	301.0356/463.0892/669.1480	quercetin-3-(2′-sinapoyl glucoside)-3′-(6′′-sinapoyl glucoside)-4′-glucoside	+				+	[26,27]
**16**	19.36	451.07916	C_27_H_15_O_7_	1.6	143.0453/128.0343	unknown	+					
**17**	20.00	299.18677	C_16_H_27_O_5_	4.9	183.1020/201.1128	1-14-dimetyl-2oxotetradecanediote	+					[31]
**18**	22.43	827.40961	C_40_H_61_O_15_	4.4	89.0230/179.0546	withanoside IV			+			[32]
**19**	22.71	421.00922	C_9_H_15_O_12_N_3_S_2_	0.3	96.9588/209.9693	unknown		+				
**20**	23.27 *	519.06006	C_22_H_21_O_6_N_3_S_3_	2.5	96.9588	unknown		+	+	+	+	
**21**	24.10	327.21771	C_18_H_31_O_9_	3.3	211.1337/229.1444	trihydroxy-octadecadienoic acid	+		+			[30]
**22**	25.29	329.21771	C_18_H_33_O_9_	4.1	211.1337/229.1444	trihydroxy-octadecanoic acid	+					[30]
**23**	26.51	811.41388	C_22_H_39_N_2_O_18_S_6_	3.2	89.0231/101.0231/179.055/221.0666	DMB-GLS			+			[17]
**24**	28.92 *	647.32922	C_31_H_51_O_14_	2.1	249.1857	unknown		+	+		+	
**25**	29.64	293.17642	C_17_H_25_O_4_	5.7	236.1053/221.1543	gingerol	+					[30]
**26**	30.43	551.03333	C_30_H_17_O_4_NS_3_	1.8	96.9589	unknown	+	+	+	+	+	
**27**	30.77 *	531.28040 [(M+FA)-H]^-^	C_25_H_41_O_9_	0.8	249.1855/253.0924	MGMG (16:3)		+	+		+	[25]
**28**	31.06 *	721.36493 [(M+FA)-H]^-^	C_33_H_55_O_14_	1.4	277.2171	DGMG (18:3)		+	+		+	[25]
**29**	33.02 *	559.31189 [(M+FA)-H]^-^	C_27_H_45_O_9_	1.0	277.2170	MGMG (18:3)		+	+		+	[25]
**30**	33.49	293.21255	C_18_H_29_O_3_	4.9	275.2018/171.1019/121.1012/293.2119	hydroxy octadecatrienoic acid	+					[33]
**31**	34.57	291.19678	C_18_H_27_O_3_	4.5	185.1177/121.1012	hydroxy octadecatetranoic acid	+					[33]
**32**	37.67	311.20145	C_13_H_27_O_8_	2.9	149.0963	unknown	+					
**33**	42.32 *	953.54846 [(M+FA)-H]^-^	C_50_H_81_O_17_	1.7	277.2172/249.1859/397.1349	DGDG (16:3,18:3)		+	+		+	[25]
**34**	44.27 *	981.57935 [(M+FA)-H]^-^	C_52_H_85_O_17_	1.2	277.2171	DGDG (18:3,18:3)		+	+	+	+	[25]

If the compound is present in several extracts of Eruca, the RT is referred to the profiles of PS, except for the compounds with *, which are referred to the profiles of BE. **Bolds numbers:** metabolites identified in freeze-dried *E. sativa* ethanolic extracts.

**Table 2:** Metabolites identified in fresh *E. sativa* ethanolic extracts by UHPLC-Q-Exactive-Orbitrap-MS/MS analysis: PS (Piana del Sele), BE (Bergamo), BR (Brescia), SP (Spontaneous), and SW (Switzerland).

N°	RT	M-H^-^	Molecular Formula	Δppm	MS/MS	Identity	PS	BE	BR	SP	SW	References
**1**	2.14	436.04074	C_12_H_22_O_10_NS_3_	1.6	96.9589/179.0173	glucoraphanin	+	+	+	+	+	[26,27]
**2**	4.22	315.07196	C_13_H_15_O_9_	2.9	152.0107/108.0206	gentisic acid glucoside	+	+	+	+	+	[28]
**3**	8.00	406.03168	C_11_H_20_O_9_NS_3_	5.4	96.9589	glucoiberverin				+		[17]
**35**	10.45 *	209.98997	C_5_H_8_O_4_NS_2_	0.9	95.9511	sulfuric acid mono[(1-thioxo-4-penten-1yl)azanyl]ester		+		+	+	[17]
**5**	14.05	385.11319	C_17_H_21_O_10_	0.7	205.0502/190.0267	sinapic acid glucoside	+		+	+		[29]
**6**	14.41 *	433.20856	C_20_H_33_O_10_	1.7	161.04445/101.0231	apigenin glucoside		+	+		+	[29]
**7**	14.76	787.1925	C_33_H_39_O_22_	−0.3	301.03571/436.0893	quercetin 3,3′,4′-triglucoside	+	+	+	+	+	[26,27]
**12**	16.22 *	609.14618	C_27_H_29_O_16_	1.2	301.0351	rutin		+	+		+	[30]
**9**	16.58 ^#^	639.15649	C_28_H_31_O_17_	0.9	476.09601/313.0359	isorhamenthin -3-4′-diglucoside			+	+	+	[26,27]
**11**	17.10	993.25079	C_44_H_49_O_26_	0.1	301.0356/463.0890/609.1470	quercetin-3,4′-diglucoside-3′-(6-sinapoyl glucoside)	+	+	+		+	[26,27]
**13**	17.57	477.06461	C_17_H_21_O_10_N_2_S_2_	1.8	96.9589/275.9957	neoglucobrassicin			+			[17]
**14**	17.85	405.02237	C_11_H_19_O_9_NS_3_	1.8	96.9589	DMB-GLS	+	+	+	+	+	[17]
**15**	18.87	1199.30701	C_55_H_59_O_30_	−1.3	301.0355/463.0890/669.1468	quercetin-3-(2′-sinapoylglucoside)-3′-(6′-sinapoylglucoside)-4′-glucoside	+	+			+	[26,27]
**36**	19.22	1169.29797	C_54_H_57_O_29_	4.5	301.0355/463.0891/639.13641	quercetin-3-(2-feruloyl-glucoside)-3′-(6-sinpoyl-glucosde)-4′-glucoside	+					[26,27]
**20**	22.47	519.05988	C_22_H_22_O_6_N_3_S_3_		96.9589	unknown	+		+	+	+	
**18**	22.58 *	827.40973	C_41_H_63_O_17_	3.8	89.03319/101.02319/179.05534	whiteside IV		+	+	+	+	[32]
**21**	24.00	327.21759	C_18_H_31_O_5_	3.0	211.1337/229.1449/171.1019	trihydroxy octadecadienoic acid	+	+	+		+	[30]
**22**	25.19	329.23294	C_18_H_33_O_5_	2.5	285.1862/211.1335/229.1444	trihydroxy octadecaenoic acid	+	+	+			[30]
**23**	26.61	811.41339	C_22_H_39_O_18_N_2_ S_6_	0.9	89.0232/119.0338/179.0552/221.0682	DMB-GLS					+	[17]
**37**	27.91 *	583.05896	C_17_H_27_O_15_N_2_S_3_	10	95.9589/178.0177/406.0309	unknown		+	+	+	+	
**26**	30.10	551.0318	C_30_H_17_O_4_NS_3_		96.9589	unknown	+	+	+	+	+	
**27**	30.70	531.28033	C_26_H_43_O_11_	0.6	249.1917	MGMG (16:3)	+			+		[25]
**28**	30.97	721.36499	C_34_H_57_O_16_	1.2	277.2175/397.1354	DGMG (18:3)	+	+	+	+	+	[25]
**29**	32.94	559.31311	C_28_H_47_O_11_	3.2	277.2175/253.0930	MGMG (18:3)	+	+	+		+	[25]
**30**	35.75 ^#^	293.17917	C_18_H_29_O_3_	3.2	275.2017/171.1011/121.1009	hydroxy octadecatrienoic acid				+	+	[33]
**32**	37.70 ^#^	311.16864	C_13_H_27_O_8_	−1.4	149.0963	unknown			+		+	
**33**	42.28 *	953.54913	C_50_H_81_O_17_	2.3	277.2172/249.18599/397.1350	DGDG (16:3; 18:3)		+	+			[25]
**34**	44.20 *	981.57965	C_52_H_85_O_17_	1.5	277.21717	DGDG (18:3; 18:3)		+	+			[25]

If the compound is present in several extracts of Eruca, the RT is referred to the profiles of PS, except for the compounds with *, which are referred to the profiles of BE, and with #, which are referred to as the profiles of SW. **Bolds numbers:** metabolites identified in fresh *E. sativa* ethanolic extracts.

The hydroalcoholic extractions, both from fresh and freeze-dried matrices, provided the most interesting results for the purpose of typifying the “Rucola della Piana del Sele” (Table 2).

**Figure 2:**
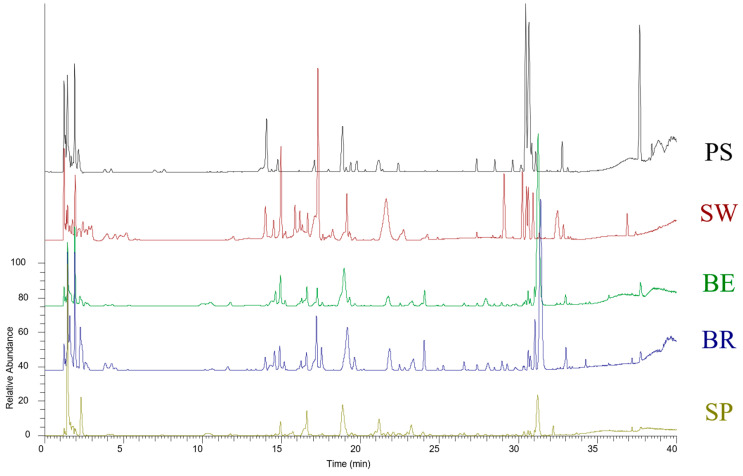
Negative profiles of fresh *E. sativa* ethanolic extracts by UHPLC-Q- Exactive-Orbitrap-MS/MS analysis: PS (Piana del Sele), BE (Bergamo), BR (Brescia), SP (Spontaneous), and SW (Switzerland).

In hydroalcoholic extracts obtained from the fresh plant, among the metabolites present in all *E. sativa* samples, was found quercetin-3,3′,4′-triglucoside (**7**), identified thanks to the fragment ion at 301 a.m.u., corresponding to the quercetin unit [20] (Figure 2). 

Other metabolites identified in each *E. sativa* sample include the glucosinolates glucoraphanin (**1**) and DMB-GLS (14) and the lipid DGMG (18:3) (**28**), due to the presence of the fragment ions digalactosyl glycerol mono-dehydrate unit at *m*/*z* 397 and RCOO fatty acyl ion at *m*/*z* 277 [25].

Glycosylated flavonoid 36 was the only metabolite identified in the “Rucola della Piana del Sele” and not present in the other samples under investigation (Figure 2).

Buitrago-Villanueva et al. identified specialized metabolites connected with a cultural system for the leaves of *E. sativa* [16]. With the aim of considering whether such metabolites are connected with geographical origin too, their content was determined in the samples of *E. sativa* harvested in different geographical areas. In particular, compound **9**, isorhamnetin 3,4 *O*-di glucoside, is present in all varieties under investigation except the *E. sativa* harvested in the “Piana del Sele” area, attesting itself as a negative marker for the differentiation of “Rucola delle Piana del Sele” from those with less market value.

Compound **11**, neoglucobrassicin, is a marker compound present only in the variety cultivated in the Brescia area.

Compound **3**, glucoiberverin, on the other hand, is a marker compound identifying the spontaneous type of Eruca sativa.

### 3.2. Multivariate Data Analysis 

The UHPLC/Q-Exactive-Orbitrap/MS row data were then subjected to a multivariate data analysis. An untargeted multivariate analysis approach was developed to distinguish between the different samples. The profiles of hydroalcoholic extracts obtained from both fresh and freeze-dried matrices of *E. sativa* were processed using the open-source software mzMine 2.10 (http://mzmine.sourceforge.net, (accessed on 23 November 2022)).

The data matrix generated by the software was then subjected to a multivariate analysis with SIMCA (+) software 12.0 (Umetrix AB, Umea, Sweden using principal component analysis (PCA) as a projection method. 

**Figure 3:**
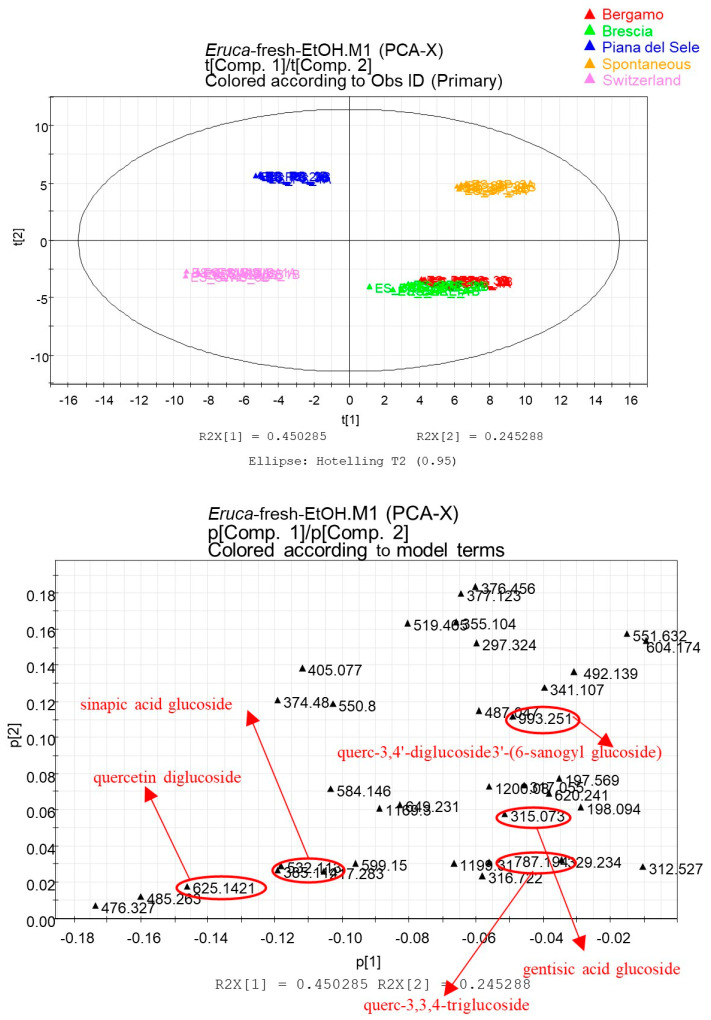
Principal component analysis (PCA) of hydroalcoholic extracts obtained from fresh samples of *E. sativa* of different geographical areas. Score scatter plot at the top and loading scatter plot at the bottom. In the loading scatter plot, biomarkers of “Rucola della Piana del Sele” are underlined.

Figure 3 shows the scatter plot representing the spatial distribution of observations (samples) for the PCA analysis of the fresh matrix extracts. The first component accounted for 45% of the variance and the second accounted for 25% of the variance.

*Eruca* samples grown in different geographical areas are well-separated within the score scatter plot. In particular, “Rucola della Piana del Sele” is separated from the others by positioning itself in the top left plot. The samples of spontaneous *Eruca* are located in the upper right plot, while those of Swiss *Eruca* are located in the lower left plot. Only the samples of *Eruca* plants grown in both Bergamo and Brescia are superimposed in the lower right part of the plot.

**Figure 4:**
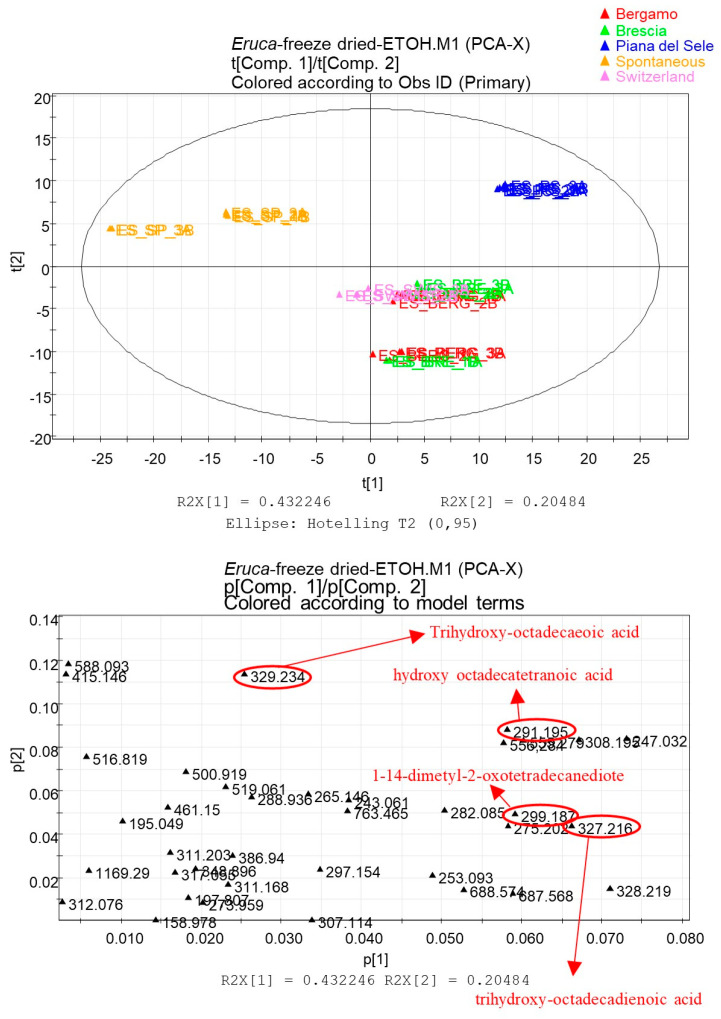
Principal component analysis (PCA) of hydroalcoholic extracts obtained from freeze-dried *E. sativa* of different geographical areas. Score scatter plot at the top and loading scatter plot at the bottom. In the loading scatter plot, biomarkers of “Rucola della Piana del Sele” are underlined.

Indeed, the loading scatter plot (Figure 3) highlights biomarker metabolites of “Rucola della Piana del Sele” that are useful for typing the PGI product. Among the metabolites that are most expressed in the *Eruca* grown in Piana del Sele, it is possible to identify glycosylated flavonoids, such as the compounds quercetin 3,3′,4′-*O*-triglucoside (**7**), quercetin-3,4′-*O*-diglucoside-3′-*O*-(6-sinapoyl-glucoside) (**10**), and quercetin diglucoside (**30**). The score scatter plot obtained from the PCA that considered samples obtained with hydroalcoholic extraction from a freeze-dried matrix shows a good clustering of the samples (Figure 4).

The first component accounted for 43% of the variance and the second accounted for 20% of the variance.

The extracts of “Rucola della Piana del Sele” are mainly grouped in the upper right part of the plot (Figure 4).

Extracts of spontaneous *E. sativa* are located in the upper left part of the plot, while extracts of *Eruca* grown in Bergamo, Brescia, and Switzerland are superimposed in the lower left part of the plot, showing a more similar metabolic expression.

The loading plot in this case confirmed the data obtained from the metabolite profiling (Figure 4).

The extracts obtained from the freeze-dried matrix of “Rucola della Piana del Sele” are rich in oxylipins. The biomarker metabolites highlighted in the loading plot are the compounds trihydroxy-octadecadienoic acid (**17**), trihydroxy-octadecaenoic acid (**18**), 1,14-dimetyl-2oxotetradecandiote (**33**), and hydroxy-octadecatretranoic acid (**37**). 

### 3.3. UPLC-ESI-QTRAP-MS/MS Quantitative Analysis

Glucosinolates (GLS) were not identified in the metabolomics approach as biomarkers. It is possible this is because GSL profiles in Brassicaceae plants can be variously affected by different parameters, but, as it is described in the literature, they are expressed in all rocket species with similar distributions. A study by Pasini et al. of 37 rocket accessions showed that their GSL profiles were all very similar [26].

Nevertheless, considering the biological interest in erucin, the myrosinase product of glucoerucin, a quantitative analytical method was developed to quantify this specific GLS in *E. sativa*, to evaluate the presence of the erucin precursor in *E. sativa* PGI and compare its content with that of *E. sativa* from other geographical origins.

Glucoerucin (GER) was quantified in the *E. sativa* samples by using a UHPLC-ESI-QTRAP MS/MS method operating in multiple reaction monitoring.

**Figure 5:**
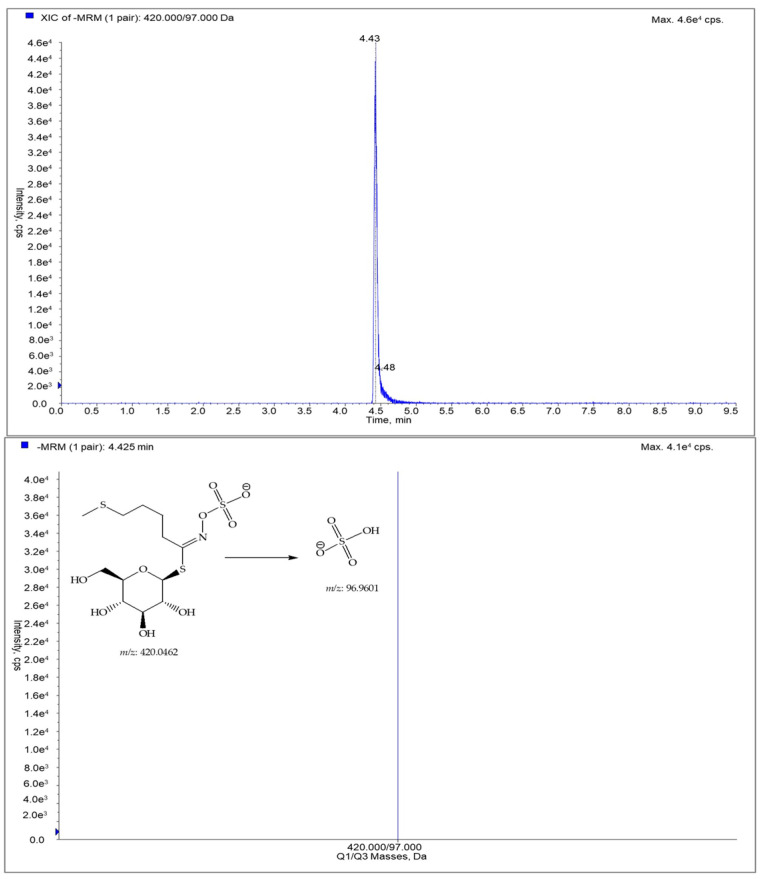
LC-ESI/QTrap/MS/MS spectrum of glucoerucin. The representative extracted ion chromatogram (XIC) of multiple reaction monitoring (MRM) chromatogram of glucoerucin and MRM MS spectrum of glucoerucin with the diagnostic fragmentation at 96.9601.

Glucoerucin displayed a pseudomolecular ion [M-H]^−^ at *m*/*z* 420, which was characterized by a diagnostic fragmentation at *m*/*z* 97 (Figure 5), corresponding to [HSO_4_]^−^ as a major product ion; consequently, this key transition was chosen for the MRM analysis, as previously suggested by Franco et al. [19].

A method validation was performed according to ICH guidelines [34], determining selectivity, the limit of detection (LOD), the limit of quantification (LOQ), linearity, and precision.

The method proved to be suitable for GER determination in complex matrices. The developed method was applied to the determination of GER in *E. sativa* extracts obtained from plants of different geographical origins.

The data were reported in Table 3 as mg of GER per g of extract obtained from different plant samples. 

A quantitative analysis showed a consistent difference in the content of GER in the different samples; in particular, *E. sativa* from Piana del Sele showed the highest content. In addition, EtOH appears to be more efficient in the extraction of this metabolite for all the samples, while the pretreatment by lyophilization affects GER content with respect to the fresh plant extraction. 

This finding is very promising for the possibility of using Rucola Piana del Sele and its wastes for the extraction of glucosinolates, and specifically for the enzymatic generation of erucin for the production of nutraceuticals for the prevention of cancer or cardiovascular diseases.

## 4. Conclusions and Discussion

A comparison of metabolite profiles did not reveal any important differences between the methanolic and hydroalcoholic extracts of *E. sativa*. These extracts were found to be rich in polyphenolic compounds such as flavonoid glycosides, particularly quercetin and kaempferol derivatives, such as quercetin-3,3′,4′-*O*-triglucoside, quercetin-3,4′-*O*-diglucoside-3′-*O*-(6-sinapoyl-glucoside), and kaempferol-3,4-*O*-diglucoside.

The glucosinolates which were most frequently detected were glucoraphanin, glucosativin, and glucoiberverin.

The analysis of dried rocket extracts from different origins showed metabolite similarity in the samples from Brescia, Bergamo, and Switzerland which were unlike the ones from “Piana del Sele”.

The peaks with the highest intensity in the profile of hydroalcoholic extracts of freeze-dried *E. sativa* PGI corresponded to oxylipins (**17**, **18**, **25**, **33**, and **37**), which could represent useful markers for the botanical origin of the plant species. These data were also confirmed by multivariate statistical analyses.

Instead, the biomarker metabolites of hydroalcoholic extracts obtained from the fresh matrix were mainly glycosylated flavonoids (**7**, **10**, and **30**).

The metabolite accumulation, such as glucosinolates and flavonols, was found to be influenced by the culture type, the harvest time, and even the growing cycle [16]. These variations constituted the criteria to choose the culture system and the harvest time of *E. sativa* leaves. It is not to be excluded that a similar variation can be expected based on the geographical origin, as observed in our experiments.

Glucosinolates (GLS) were not identified in the metabolomics approach as biomarkers, although it is well known that GSL concentrations can vary and change over time depending on environmental conditions [35]. GSL profiles in Brassicaceae plants in fact vary, being affected by different factors, including plant age, organ type, developmental stage, ambient air temperature, level of water stress, photoperiod, agronomic practice, degree of wounding, and geographical origin of the variety/species [36]. On the other hand, glucosinolates are expressed in all rocket species with similar distribution. A study by Pasini et al. of 37 rocket accessions showed that GSL profiles were all very similar, regardless of the cultivar [26]. 

In conclusion, metabolite profiling, through UHPLC-Q-Exactive-Orbitrap-MS/MS analysis, is useful for the typization of *Eruca sativa* “Rucola della Piana del Sele” (PGI). 

Furthermore, *Eruca sativa* cultivated in the Piana del Sele showed the highest value of GER, with a promising ability to generate erucin through its conversion by myrosinase action.

Both of the approaches (untargeted metabolomics and glucoerucin determination) can be actually applied for the typization of the PGI *E. sativa*, with better performance in a combined untargeted/targeted approach.

Future perspectives could be oriented to improving the metabolomics approach, extending the analysis to volatilomics, to evaluate if there is a specific contribution to the differentiation of these compounds, for a comprehensive point of view. 

## Figures and Tables

**Table 3:** Quantitative results of glucoerucin occurring in EtOH and MeOH extracts of *E. sativa* leaves.

	µg/g Extract of *E. sativa* Fresh Leaves ± SD *	
	EtOH Extracts	MeOH Extracts
PS	688.33 ± 11.55	341.67 ± 6.40
BE	60.00 ± 1.80	50.67 ± 0.58
BR	44.88 ± 2.35	57.67 ± 0.29
SP	57.67 ± 0.29	191.17± 4.62
SW	28.57 ± 0.12	43.90 ± 2.60
	µg/g extract of *E. sativa* freeze-dried leaves ± SD *	
PS	61.67 ± 2.47	52.83 ± 2.57
BE	38.73 ± 0.46	39.37 ± 1.96
BR	74.50 ± 3.46	48.35 ± 2.25
SP	58.83 ± 1.15	166.17 ± 10.10
SW	47.47 ± 1.79	41.93 ± 1.76

* Standard deviation of three independent experiments.

## Data Availability

The data used to support the findings of this study can be made available by the corresponding author upon request.
